# Green synthesis, characterization, antioxidant, antibacterial, and photocatalytic activity of *Suaeda maritima* (L.) Dumort aqueous extract-mediated copper oxide nanoparticles

**DOI:** 10.1186/s43141-021-00229-9

**Published:** 2021-08-30

**Authors:** Pavani Peddi, Prasada Rao PTSRK, Nannapaneni Usha Rani, S. Lakshmi Tulasi

**Affiliations:** 1 Department of Chemistry, Prasad V Potluri Siddhartha Institute of Technology, Kanuru, Vijayawada, AP 520007 India; 2 Department of Chemistry, P.B. Siddhartha College of Arts and Science, Vijayawada, AP 520010 India

**Keywords:** Green synthesis, Copper nanoparticles, *Suaeda maritima*, Pharmacological activity, Photocatalytic activity

## Abstract

**Background:**

The aim of this work was to synthesize copper oxide nanoparticles (CuO NPs) utilizing heartwood aqueous extract of *Suaeda maritima* (L.) Dumort. The synthesis of CuO NPs using green methodology with small size and high stability paved the way to protect the environment by not involving toxic chemicals and environment-friendly methodology for pharmacological and photocatalytic applications. The aqueous areal parts extract of *S. maritima* (L.) Dumort was used for synthesis, characterization of CuO NPs was studied, and further its antioxidant, antibacterial, and photocatalytic activity for the removal of methylene blue was studied.

**Results:**

The synthesized CuO NPs shows characteristic UV-visible absorption maximum at 282 nm. The FT-IR spectra shows peak at 3640 cm^−1^ attributed to hydrogen bonded O-H group of poly phenols, alcohols, and N-H of amide. Strong peak at 1122 cm^−1^ corresponds to C-OH stretch in phenols and alcohols. Peaks at 1467 cm^−1^ and 1585 cm^−1^ corresponds to C=C in aromatic compounds. Strong peak at 1749 cm^−1^ represents the C=O in aldehydes or in keto compounds. Several strong bonds identified in the range of 1088 to 1225 cm^−1^ representing C-O-C stretch vibrations. The synthesized particles were circular in shape with rough surface morphology and dispersed as clusters with size of 37 nm with metallic content of 73.8%. The synthesized CuO NPs were proved as potent antibacterial and antioxidant activities. The photocatalytic for the removal of methylene blue in aqueous solution was studied and results proved that the CuO NPs were effectively remove the dye up to 86.91% within less time of 75 min. Hence, the CuO NPs synthesized are high efficiency with less particle size and can be used as antioxidant, antibacterial agent, and also applicable for the removal of hazardous methylene blue dye from effluents and can contribute indirectly to clean up the environment.

**Conclusions:**

The investigation reports the eco-friendly, cost-effective method for synthesizing copper oxide nanoparticles from *S. maritima* extract with biomedical applications.

## Background

In recent years, nanoparticles (NPs) technology plays significance role in the field of medical, pharmaceutical, and textile industries [1]. NPs and nanomaterials were used in different purposes like diagnosis, targeted drug delivery, cosmetics, biosensor, and many more. Metal NPs have different size, shape, composition, and exhibit different and unique physico-chemical properties. In view of this, different researchers around the world are exploring the versatile characteristics and applications of NPs [2, 3]. Metal NPs such as copper, tin, iron, zinc, and nickel have remarkable applications in biomedical, energy, and environment fields of study [4].

Silver, zinc, gold, etc. metal NPs are having remarkable applications in medical institutes for some years [5]. NPs were exhibit strong adsorption capability which is responsible for its enriched performance and applications [6]. Metallic NPs having various morphologies and sizes can be synthesized using different physical and chemical methods. These methods involve non-standard procedure, are difficulty to perform, and are expensive. Most of the chemical-mediated NP synthesis utilize toxic chemicals such as reducing agents, non-biodegradable stabilizing agents, and organic solvents. These chemicals are dangerous to biological, aquatic systems and the environment [7]. Thus, the synthesis of NPs using biological methods is proved as environmental friendly and economical and is served as the best alternative method for synthesis of NPs. In biological NP synthesis methods, microbes and bioactive plant extracts are used as reducing agent. The growing of microbial culture for a long period is very difficult and there is a possibility of contamination and hence use of plant bioactive compounds for the NP synthesis is very simple, economical, and convenient [8].

In recent days, copper (Cu), nickel (Ni), zinc (Zn), etc. are used for NP synthesis instead of noble metals such as gold and silver because noble metals are rare and high cost. Copper oxide (CuO) NPs have a wide range of application in different fields and are used in catalytic [9], optical [10], superconductor [11], and magnet resistance materials [12] and solar energy transformation [13] applications. CuO NPs also having antimicrobial [14], antidiabetic, anti-cancer [15], and biocidal [16] properties. CuO NPs are nontoxic and having antimicrobial efficacy in controlling plant diseases, photocatalytic activity for dye effluent treatment, and many other environmental applications [17].

The *Suaeda maritima* (L.) Dumort., belongs to the family Amaranthaceae, commonly known as seablite and locally called as Elakura in Andhra Pradesh. It is grown in coastal salt flats and tidal wetlands near the sea and is distributed worldwide. It is edible as a leaf vegetable and used for making juice and curries, feeding cattle, goats, and sheep [18]. In local traditional medicine, it is used for the treatment of hepatitis and is having hepatoprotective [19], antioxidant [20], and antimicrobial [21] activities.

In the literature survey, to the best of our knowledge, there are no reports available for the synthesis of CuO NPs using any part of the plant *S. maritima*. In view of the above, the present investigation is focused on the synthesis and characterization of CuO NPs using aqueous whole plant extract of *S. maritima*. In addition, antibacterial activity and DPPH radical scavenging activity of the synthesized CuO NPs were studied.

Methylene blue is a heterocyclic aromatic compound and a cationic dye, widely used for dying cotton, wool, and silk. The harmful effect of the existence of this dye in waste water may have arisen from the burns effect of eye, nausea, vomiting and diarrhea, etc. It may be poisonous if it is inhaled and in contact with skin. Thus, it is necessary to remove such a hazardous dye from industrial effluent before it pollutes the nearby freshwater streams. Hence, in the present study, the application of the synthesized CuO NPs for the photocatalytic degradation of methylene blue was also investigated.

## Methods

### Materials

Copper (II) sulfate penta hydrate, 2,2-diphenyl-1-picrylhydrazyl (DPPH), peptone, beef extract, agar, methylene blue, and sodium hydroxide were purchased from Merck Chemicals, Mumbai. The areal parts of the plant *S. maritima* (L.) Dumort., was collected in mangrove forest, near Gilakaladindi, Machilipatnam, Krishna District, AP. The field studies were conducted in accordance with the local legislations and have taken necessary permissions. The collected areal plant parts were cleaned, shade dried, powdered, and used for CuO NP synthesis.

### Preparation of plant extract

Two grams of dried plant powder was added in 200 mL distilled water in a 500-mL flask, mixed well on a magnetic stirrer with hotplate at 60 °C for 20 min. Then, it was filtered using Whatman #1 paper and the filtrate was preserved for NP synthesis and also for the evaluation of phytochemical constituents in the aqueous plant extract by preliminary screening tests as per reported methods [22].

### Synthesis of CuO NPs

The one pot green synthesis of CuO NPs was performed as per the procedure available in literature [23, 24] briefly, to 50 mL of 5 mM copper sulfate solution, 5 mL of extract was added and pH was adjusted to 7 using 1 N sodium hydroxide solution. The color changed to green color and the solution was centrifuged and pellet was dried in air oven at 60 °C for 24 h. A dark brown/black color powder was obtained and was stored in room temperature for further study.

### Characterization of CuO NPs

The double beam UV-visible spectrophotometer (JASCO, Japan) was used for determination of optical absorption of CuO NPs in the wavelength region of 800 to 200 nm. The nature of the bioactive compounds involved in the bio-reduction of Cu was identified by performing functional group identification on Fourier transform infrared spectroscopy (FT-IR, Bruker, USA) which is performed in 4000 to 500 cm^−1^ range. FE-SEM (field emission scanning electron microscope — Nova, Nanosem-450, FEI, USA) study was carried for the determination of morphology and size of the synthesized NPs. X-ray diffractometer (Rigaku Corporation) was studied for the determination of crystalline and lattice structure of the NPs and is carried at a scan speed of 2°/min in the diffraction angles (2θ) from 20 to 80°. Energy-dispersive X-ray spectroscopy (RONTEC’s EDX system, Model QuanTax 200, Germany) studies were carried for the determination of elemental composition of the synthesized NPs. The zeta potential and size distribution of the NPs were determined using dynamic light scattering (DLS) technology using Malvern Zetasizer (Nano ZS90, UK) at 25 °C, at an angle of 17 °C and 78.5 dielectric constant [25, 26].

### Antibacterial activity of synthesized CuO NPs

The antibacterial activity of synthesized NPs was carried against two gram-positive and two gram-negative bacteria namely *Bacillus subtilis* (MTCC — 1427) and *Staphylococcus aureus* (MTCC — 1430) and two gram-negative bacteria namely *Escherichia coli* (MTCC — 294) and *Pseudomonas aeruginosa* (MTCC — 1748) using well diffusion method on nutrient agar plate as per the procedure described by Priyanka et al. [27]. In a sterile petri dish, 10 mL of nutrient agar medium was poured as a basal layer followed with 15 mL of seeded medium previously inoculated with selected bacterial suspension (100 mL of medium/1 mL of 10^7^ CFU) to attain 10^5^ CFU/ml of medium. Then, wait till the complete solidification of the medium in the petri plate and wells were prepared using sterilized stainless-steel cork borer. In each well, 25 μL of selected concentration of aqueous plant extract, NPs solution, and Gentamycin (standard) were loaded with a sterile micro-pipette. Simultaneously in a separate petri dish, water was loaded and served as negative control and plates were grown at 37 °C for 24 h. Then, the zone of inhibition of standard, CuO NPs. and aqueous plant extract were measured in millimeters by comparing with negative control.

### DPPH radical scavenging assay of synthesized CuO NPs

The DPPH free radical scavenging assay of synthesized CuO NPs was carried out by the method of described by Thirunavukkarasu et al. [28]. In 1 mL of 0.135 mM methanolic DPPH solution, 1 mL of different concentrations of synthesized CuO NPs and aqueous plant extract were added separately and the reaction mixture was incubated in room temperature for 30 min. The absorbance of the resultant solution was measured using UV-visible spectrophotometer at 517 nm. The DPPH inhibition activity of the synthesized nanoparticles was calculated using the resultant absorbance vales with that of the control values.

### Photocatalytic degradation of methylene blue

Standard methylene blue solution at a concentration of 50 μg/mL and 100 μg/mL was selected for the photocatalytic efficiency *S. maritima* (L.) Dumort mediated CuO NPs. The standard dye solution was treated with different strengths of synthesized CuO NPs and was kept in sunlight. Then, with an interval of every 1 h, 2.0 mL of dye solution was taken and centrifuged at 3000 rpm for 5.0 min. The supernatant solution absorbance was determined by UV-visible spectrophotometer at 664 nm and the % dye present in the solution and the % dye degraded due to the treatment with CuO NPs was calculated using standard calibration curve [29].

## Results

The phytochemical analysis results of *S. maritima* (L.) Dumort aqueous extract was given in Table 1.

**Table 1:** Phytochemical screening results of areal parts aqueous extract of Suaeda maritima (L.) Dumort

S No	Phytochemicals	Results observed for ***S. maritima*** aqueous extract
1	Alkaloids	-
2	Flavonoids	+++
3	Phenolic compounds	+
4	Terpenoids	++
5	Steroids	-
6	Cardiac glycoside	++
7	Proteins	+
8	Carbohydrates	+
9	Amino acids	-
10	Saponins	++

“+++”, immensely present; “++”, moderately present; “+”, slightly present; “-” absent

**Fig. 1:**
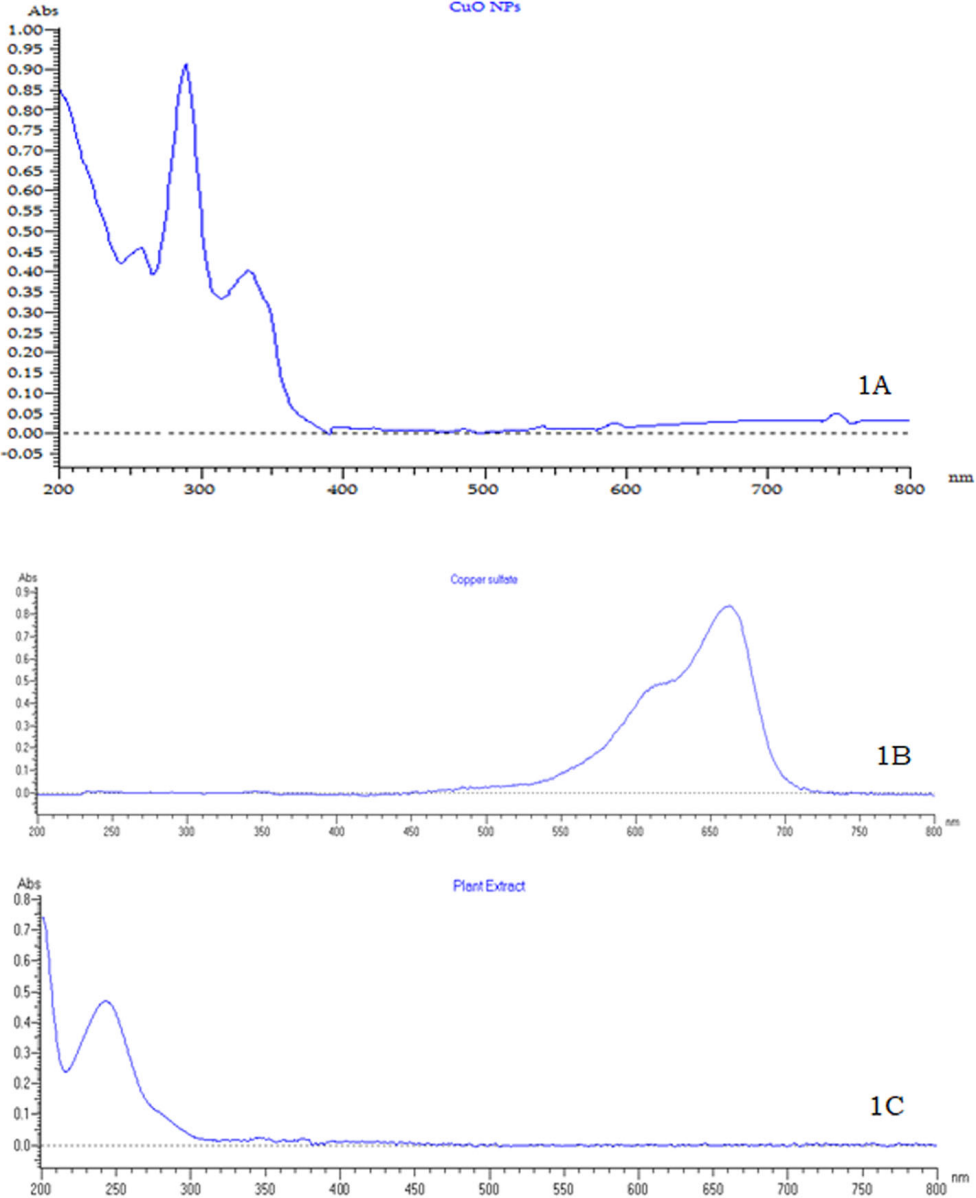
UV-visible absorption of spectra of CuO NPs synthesized using *S. maritima* extract (**A**), copper sulfate aqueous solution (**B**), and aqueous plant extract (**C**)

The aqueous extracts of areal parts of plant *S. maritima* (L.) Dumort was used for the synthesis of CuO NPs. The formation of green color may be surface plasmon vibrations with copper oxide nanoparticles which confirms the bio-reduction of copper and the formation of CuO NPs. The bio-reduction of copper and the formation of NPs were checked using UV-visible spectrophotometer (Fig. 1).

**Fig. 2:**
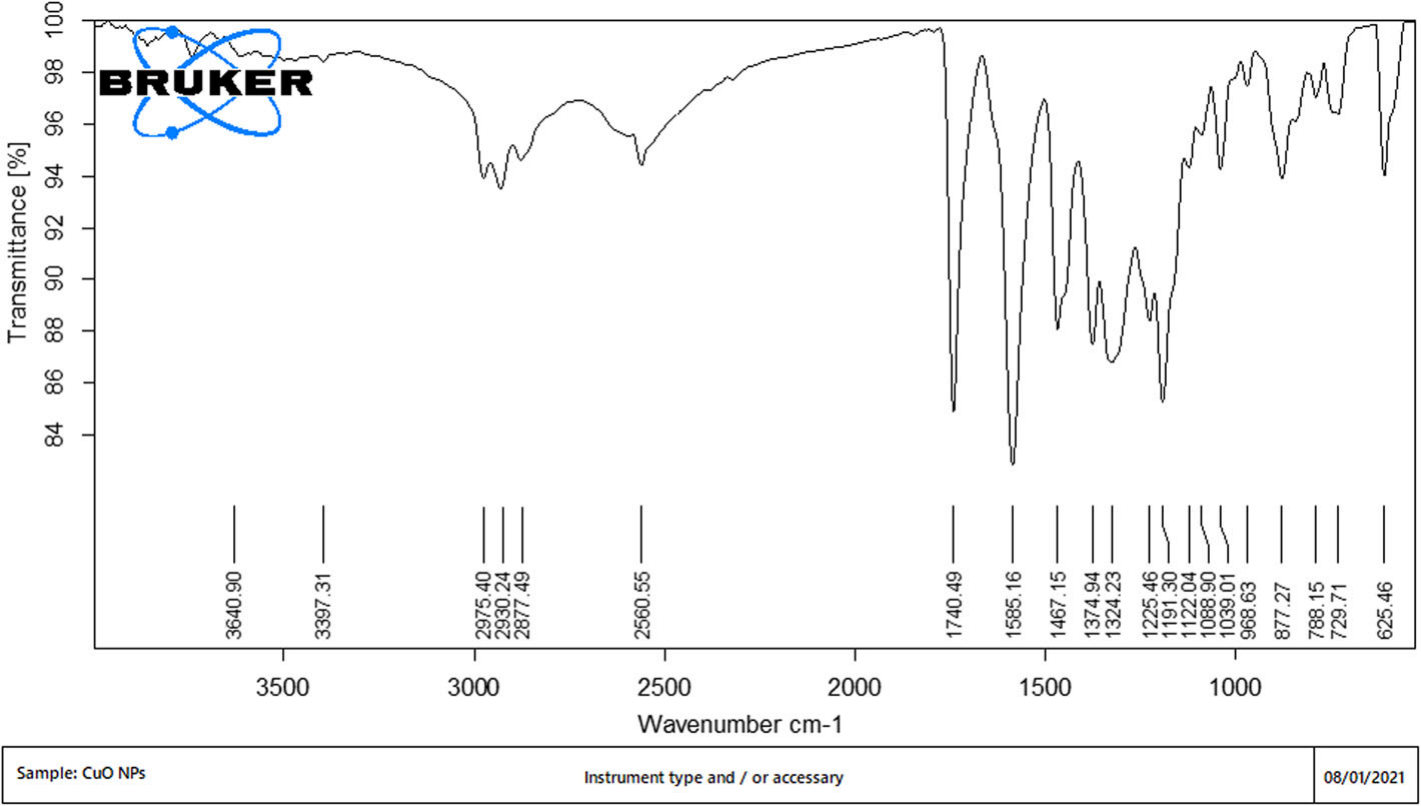
FT-IR analysis spectrum of *S. maritima* (L.) Dumort aqueous areal parts extract based CuO NPs

The surface analysis of CuO NPs synthesized using *S. maritima* (L.) Dumort as bio-stabilizing agent gives an idea about the involvement of biomolecules that are responsible for the reduction and the capping of nano-composites. The FT-IR spectra of the CuO NPs (Fig. 2) shows absorption peaks due to bio molecules present in the plant extract and reflects the complex nature of the NPs. The FT-IR spectra shows peak at 3640 cm^−1^ attributed to hydrogen bonded O-H group of poly phenols, alcohols, and N-H of amide. Strong peak at 1122 cm^−1^ corresponds to C-OH stretch in phenols and alcohols. Peaks at 1467 cm^−1^ and 1585 cm^−1^ corresponds to C=C in aromatic compounds. Strong peak at 1749 cm^−1^ represents the C=O in aldehydes or in keto compounds. Several strong bonds identified in the range of 1088 cm^−1^ to 1225 cm^−1^ representing stretch vibrations in C-O-C bond.

**Fig. 3:**
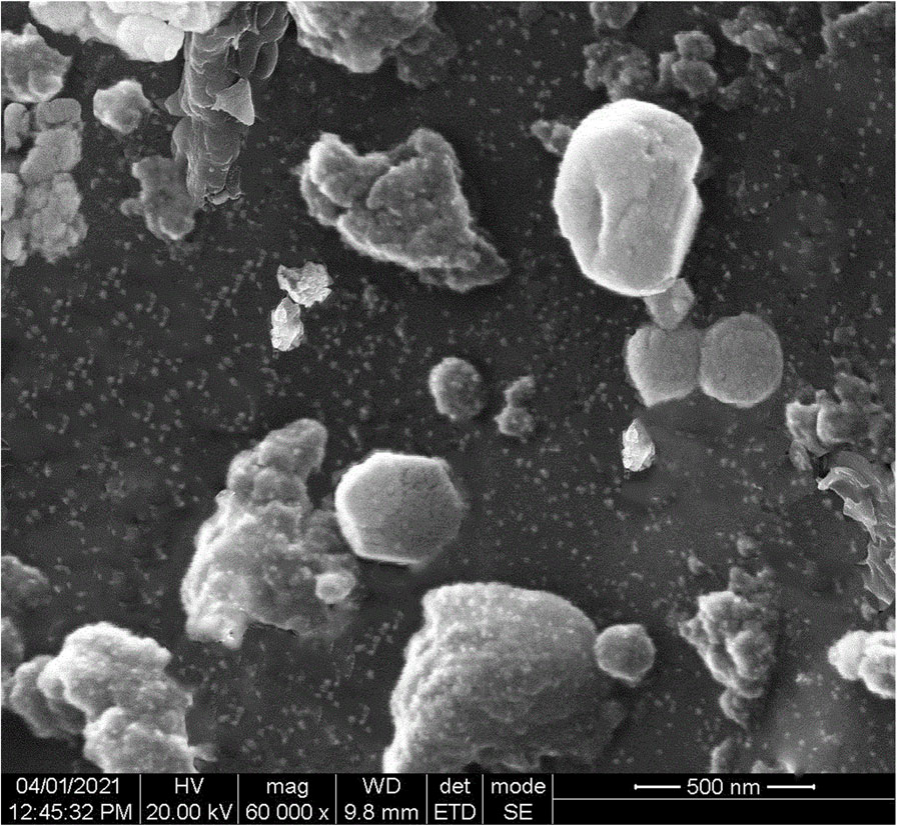
SEM photo of *S. maritima* (L.) Dumort aqueous areal parts extract based CuO NPs

The micrograph observed in SEM analysis of synthesized CuO NPs is shown in Fig. 3. The SEM micrograph shows agglomerations of the NPs and the shape of the NPs was observed to be circular with rough surface morphology and dispersed as clusters. The size of the obtained CuO NPs was in 10–60 nm size range.

**Fig. 4:**
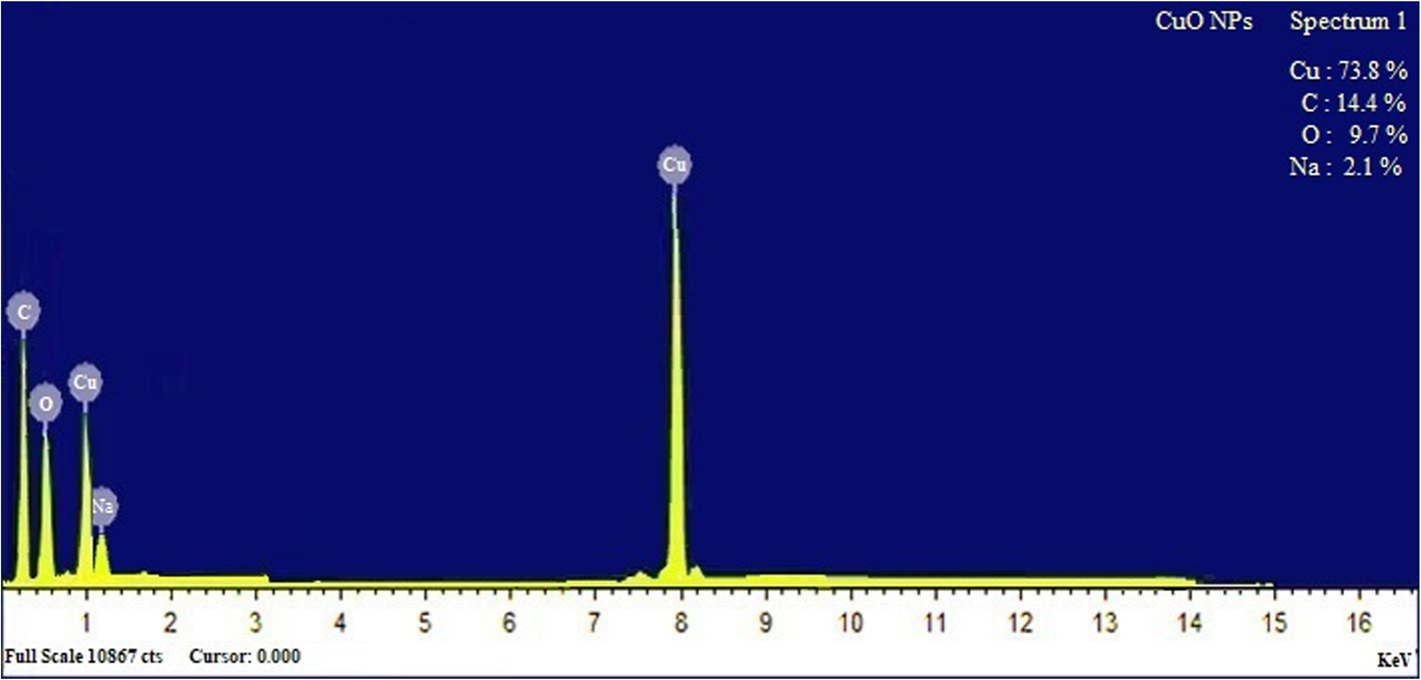
EDX profile of CuO NPS

The elemental composition of *S. maritima* (L.) Dumort extract-mediated CuO NPs was confirmed by EDX analysis and the spectrum was given in Fig. 4.

**Fig. 5:**
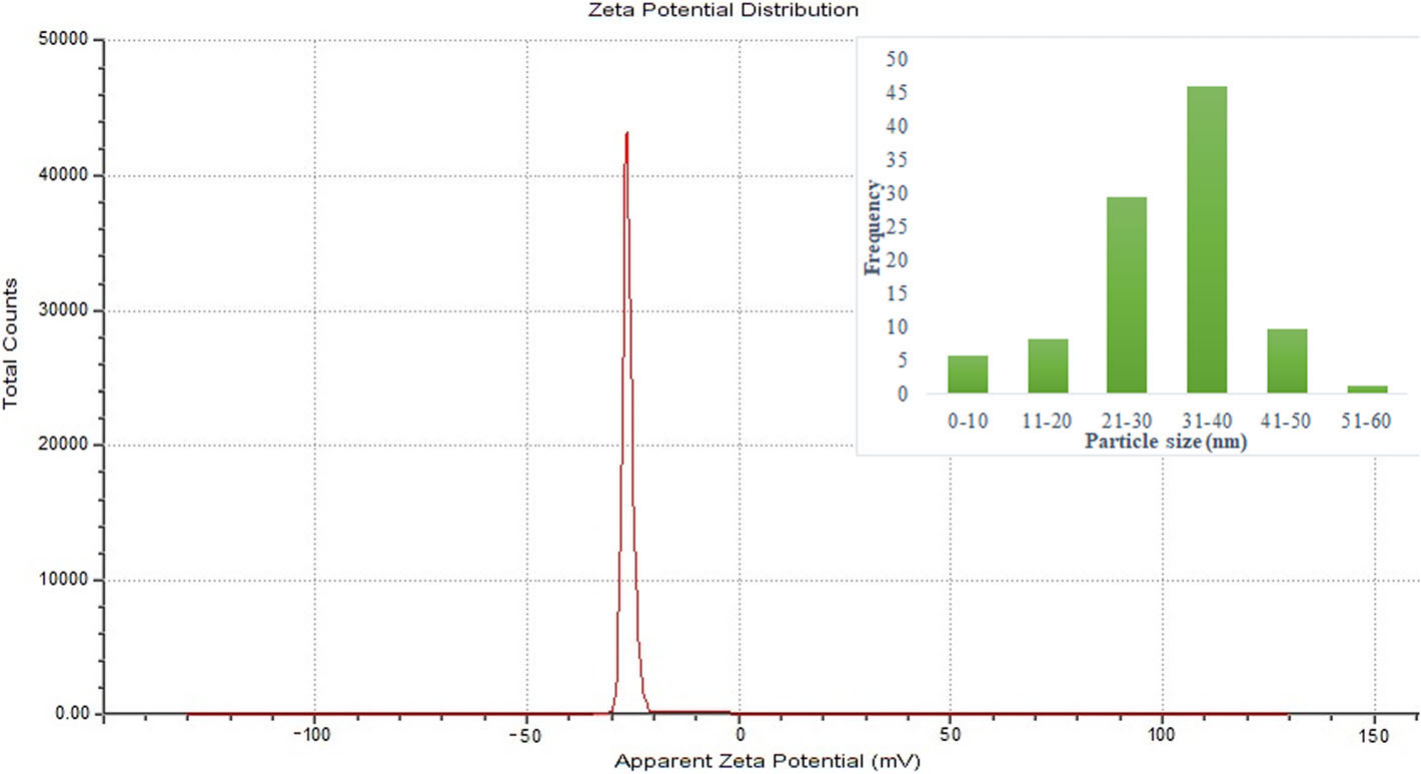
Zeta potential and particle size distribution of CuO NPs

Dynamic light scattering (DLS) technology is used for the determination of particle size distribution of synthesized CuO NPs. The results found that CuO NPs were distributed in the size of less than 60 nm. The maximum abundance of size distribution was observed in 21–40 nm. The less than 10 nm and greater than 50 nm size particles were very less and having 37 nm as average size of the particles and is further confirmed using XRD analysis. The DLS technology is also utilized for evaluation of zeta potential of CuO NPs. The results show that the CuO NPs were a negative change and was observed at 26.6 mV (Fig. 5). The high negative zeta potential causes strong repulsion between the particles [24] and the particles formed in this study was found to be stable.

**Fig. 6:**
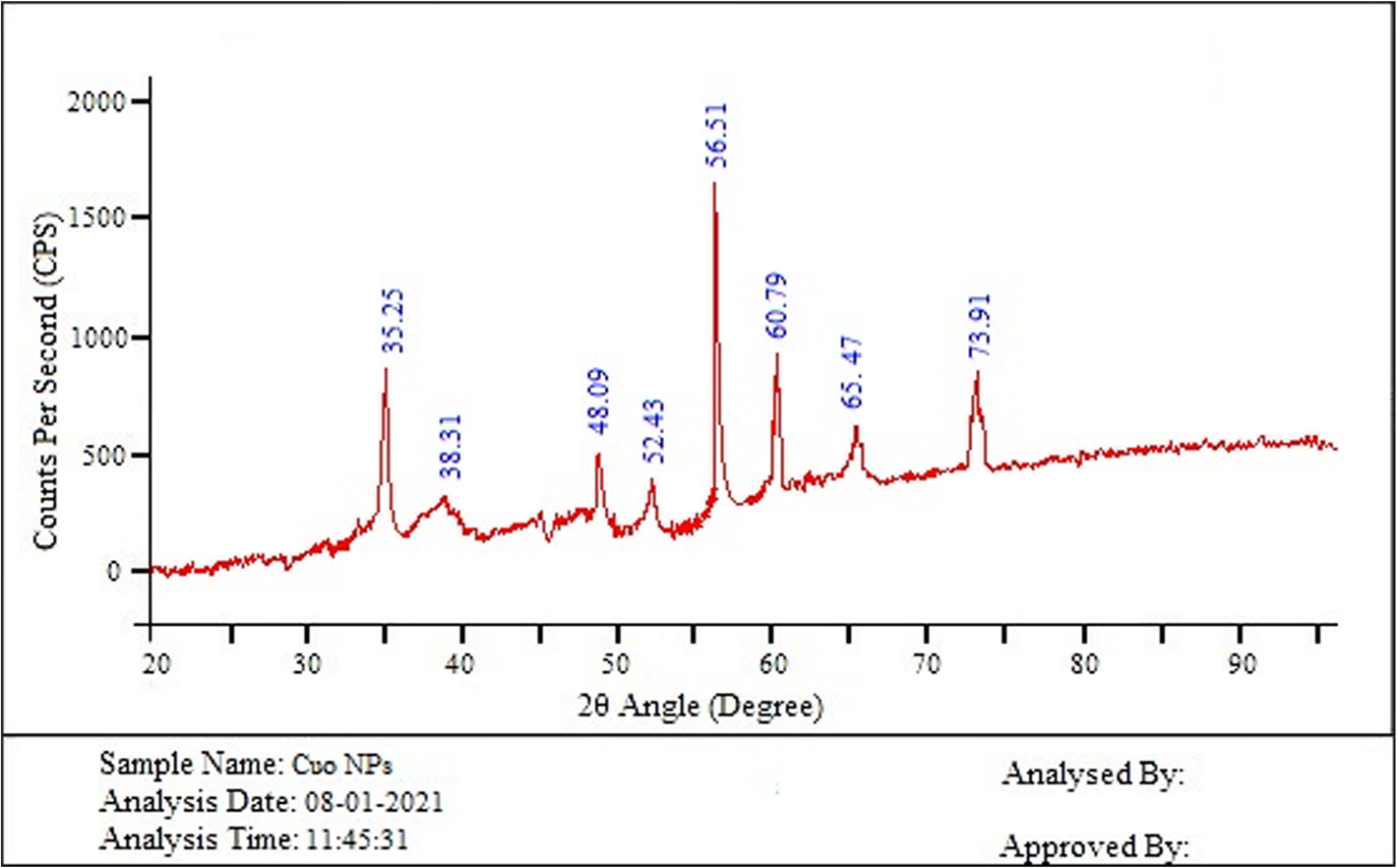
XRD spectra of CuO NPs Antibacterial activity study

The crystalline nature and phase orientation of the synthesized CuO NPs was determined using XRD studies. The XRD pattern shows the peaks position with 2θ values of 35.25°, 38.31°, 48.09°, 52.43°, 56.51°, 60.79°, 65°, 47°, and 73.91° (Fig. 6) are indexed as (002), (111), (202), (020), (202), (113), (311), and (113) planes. From the XRD spectra, the interplanar spacing and lattice parameter values were calculated and was found to be 0.2269 nm and 0.3983 nm respectively which are in correlation with the standard International Center of Diffraction Data card (JCPDS-45-0937) for CuO NPs confirms that the formed NPs are in crystalline nature with an average particle size of 34 nm.

**Fig. 7:**
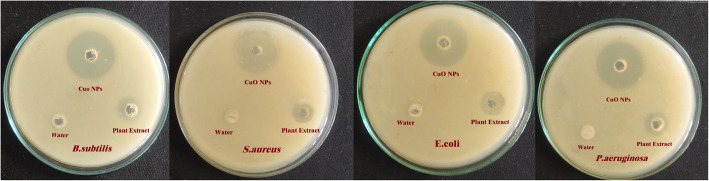
Images of antibacterial activity results of aqueous extract of *S. maritima* (L.) Dumort and its mediated CuO NPs

**Fig. 8:**
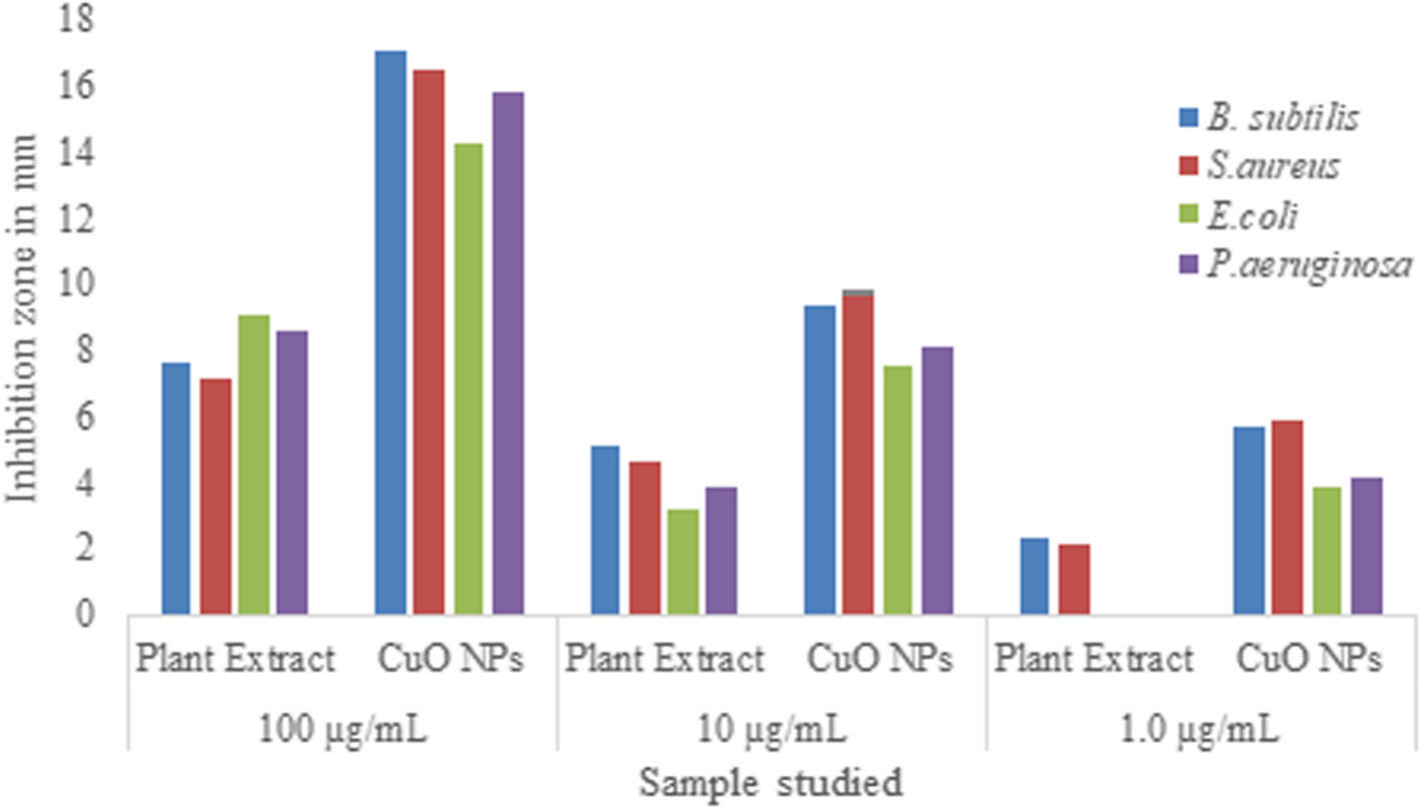
Comparison of images of antibacterial activity of aqueous plant extract of Suaeda maritima (L.) Dumort and its mediated CuO NPs

The synthesized CuO NPs show potential growth inhibition against bacterial strains studied. The results of the antibacterial activity confirm that the activity was enhanced for the synthesized CuO NPs than water extract and the results were given in Table 2; zone of inhibition was represented in Fig. 7 and the graphical comparison of different studied concentrations against bacterial strains was shown in Fig. 8.

**Table 2:** Antibacterial activity results of aqueous extract of *S. maritima* (L.) Dumort and its mediated CuO NPs

S No	Organism studied	Sample at 100 μg/mL concentration of		Sample at 10 μg/mL concentration of		Sample at 1.0 μg/mL concentration of	
		Plant extract	CuO NPs	Plant extract	CuO NPs	Plant extract	CuO NPs
1	*B. subtilis*	7.6	17.1	5.1	9.4	2.3	5.7
2	*S. aureus*	7.1	16.5	4.6	9.8	2.1	5.9
3	*E. coli*	9.1	14.3	3.2	7.5	0	3.9
4	*P. aeruginosa*	8.6	15.8	3.9	8.1	0	4.2

Values given in the table are the average of three replicate experiments

**Fig. 9:**
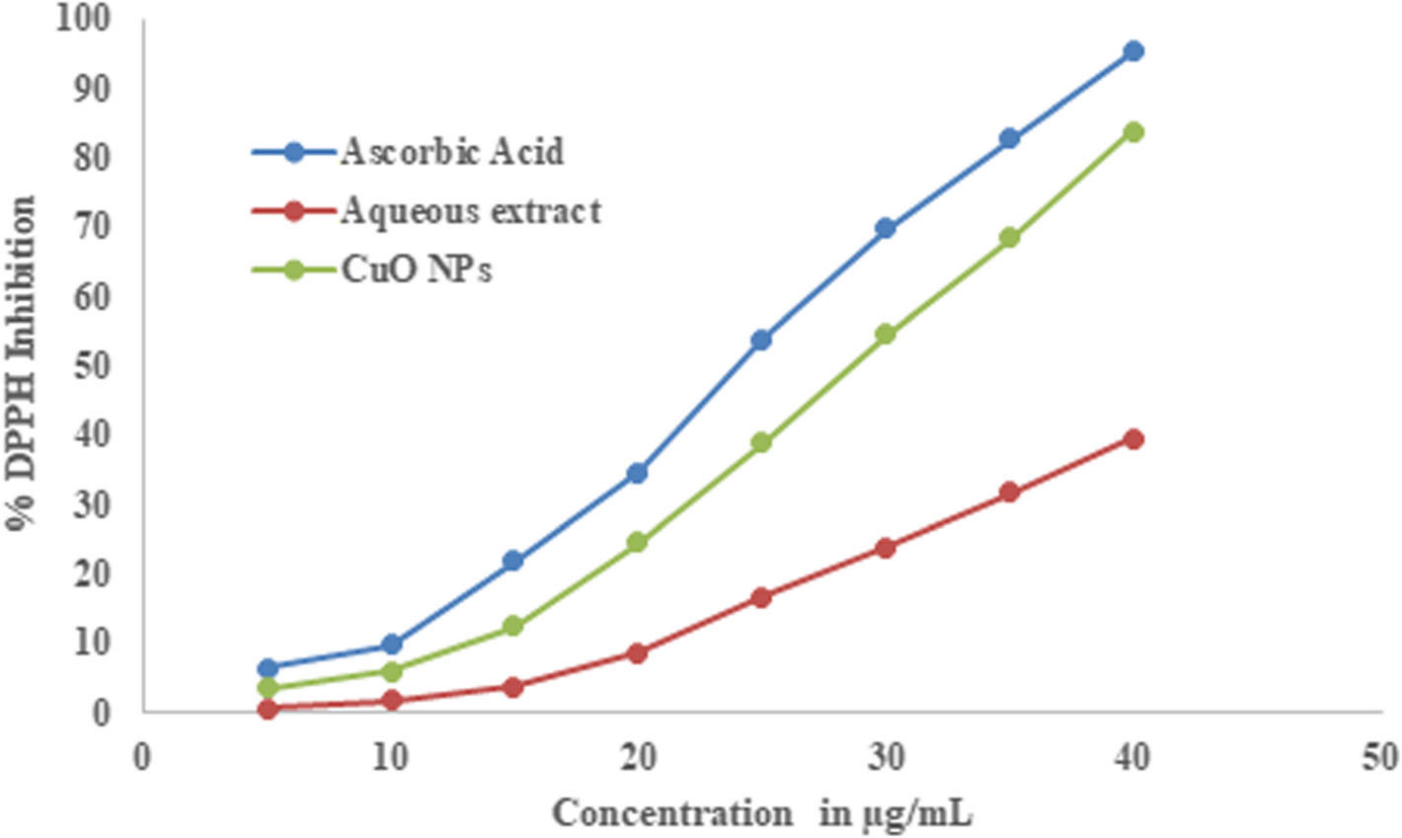
Comparative graph of DPPH assay study results of standard ascorbic acid, aqueous extract of *S. maritima* (L.) Dumort, and Cuo NPs synthesized in the study

The DPPH free radical assay of CuO NPs and the aqueous plant extract was compared with ascorbic acid (standard) and aqueous plant extract. It was obtained that both plant aqueous extract and the synthesized CuO NPs having DPPH inhibition activity and are compared with that of standard ascorbic acid. The IC 50 of standard ascorbic was found to be 23.67 μg/mL, whereas the green synthesized CuO NPs was found to be 28.05 μg/mL which is very close to the standard and the aqueous plant extract has 51.71 μg/mL. The results confirm that CuO NPs have enhanced radical inhibition activity than aqueous extract and are very close to standard. The results of DPPH inhibition study were given in Table 3 and the comparison graph was represented in Fig. 9.

**Table 3:** Results obtained in DPPH radical scavenging assay

S No	Concentration in μg/mL	% DPPH Inhibition		
		Ascorbic Acid	Aqueous extract	CuO NPs
1	5	6.52	0.57	3.51
2	10	9.81	1.62	5.93
3	15	21.89	3.71	12.55
4	20	34.69	8.56	24.64
5	25	53.91	16.83	38.93
6	30	69.73	23.88	54.67
7	35	82.68	31.69	68.44
8	40	95.21	39.52	83.91

Values given in the table are the average of three replicate experiments

### Photocatalytic degradation of methylene blue

**Fig. 10:**
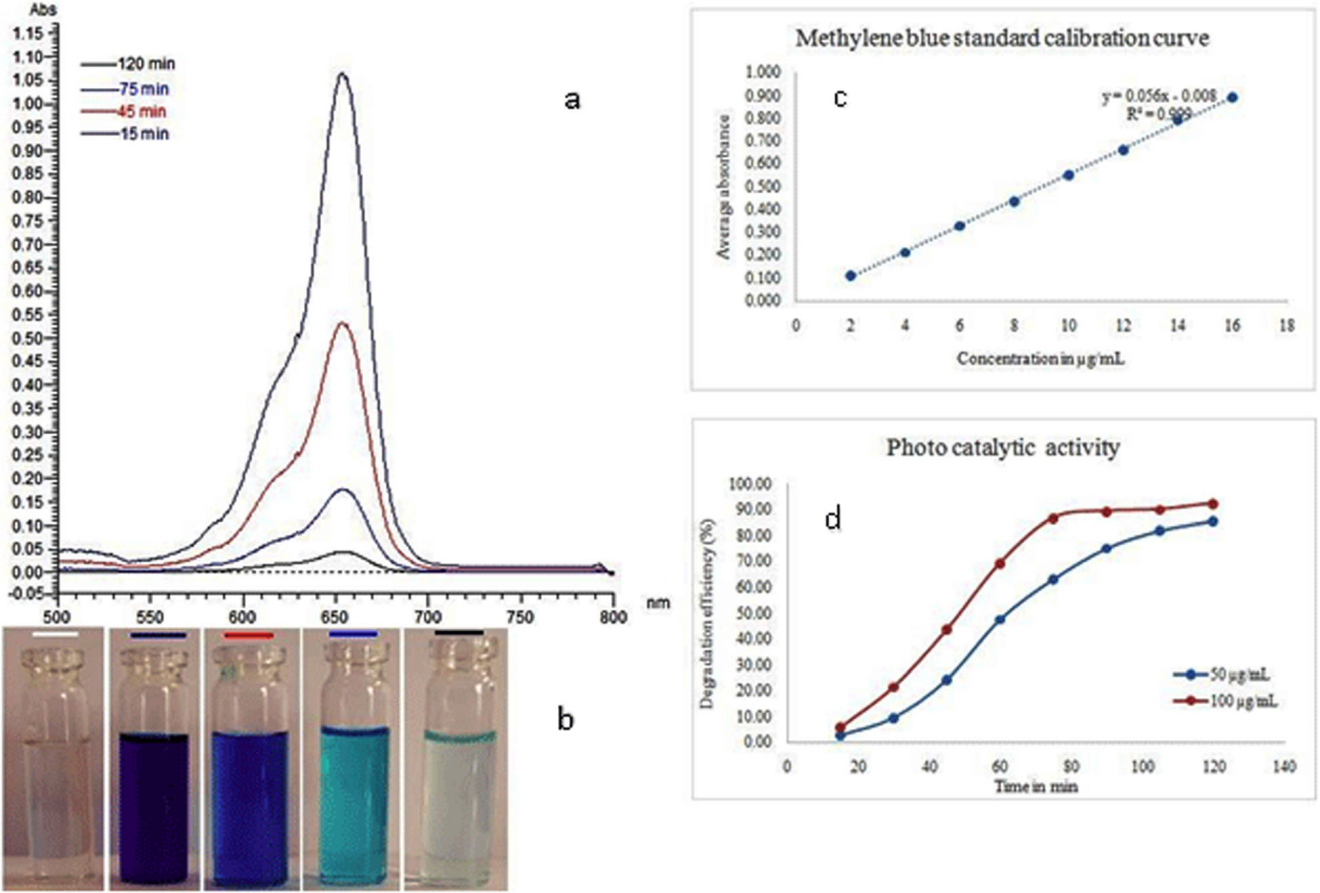
Methylene blue degradation using Cuo NPs synthesized using aqueous extract *S. maritima* (L.) Dumort. **a** Methylene blue treated with Cuo NPs showing absorption at different time intervals using UV-visible spectrophotometer. **b** Color of the methylene blue in different time intervals. **c** Methylene blue standard calibration constructed for the estimation in each time interval. **d** Photocatalytic activity of CuO NPs at 50 μg/mL and 100 μg/mL concentration of methylene blue

The photocatalytic activity of CuO NPs was studied for the degradation of methylene blue dye using UV-visible spectrophotometer at 664 nm. The activity was confirmed by carrying a control experiment without NPs. It was confirmed that the dye was kept in sunlight without CuO NPs; there is no change in the strength/concentration and remarkable decrease in strength of dye in presence of CuO NPs (Fig. 10a) and reached minimum at time interval of 120 min (Fig. 10b). The concentration of methylene blue dye in each time interval was calculated using standard calibration curve (Fig. 10c). At a concentration of 50 μg/mL of methylene blue, the % degradation was less and at a concentration of 100 μg/mL, the % degradation was high (Fig. 10d). The results of photocatalytic degradation of methylene blue by Cuo NPs synthesized using *S. maritima* (L.) Dumort aqueous extract was shown in Table 4.

**Table 4:** Methylene blue photocatalytic activity results of CuO NPs synthesized using aqueous extract of *S. maritima* (L.) Dumort

S No	Time in min	% Photocatalytic degradation at a concentration of	
		50 μg/mL	100 μg/mL
1	15	2.84	5.93
2	30	9.73	21.58
3	45	24.45	43.71
4	60	47.61	69.52
5	75	63.22	86.91
6	90	75.13	89.57
7	105	82.01	90.23
8	120	85.70	92.55

Value given in the table are the average of three replicate experiments

## Discussion

The UV-visible absorbance spectra show wavelength maxima centered near 282 nm (Fig. 1A) indicating the reduction of copper sulfate to CuO NPs. The similar type of UV absorption peak was reported in the literature and provides correlation with the present study [30]. There is no absorption peak in the region of 282 nm observed in the UV spectra of aqueous copper sulfate solution (Fig. 1B) and aqueous plant extract (Fig. 1C) confirms that the absorption peaks at 282 nm is due to the formation of CuO NPs. In the UV-visible spectra, there is another peak at 328 nm was observed and this may be due to the presence of bioactive compounds in the plant extract that are responsible for the formation of CuO NPs.

The FT-IR analysis of CuO NPs confirms that flavonoids, phenolic compounds, terpenoids, cardiac glycoside, and saponins are the chemical constitutes shows positive in the preliminary screening of the aqueous extract of *S. maritima* may be acts as reducing, stabilizing, and dispersing agent for CuO NPs formation. In addition to the plant-based bioactive compounds, the characteristic vibrational peak corresponding to CuO NPs was observed at 625 cm^−1^ conforms the formation of copper NPs [31, 32]. The SEM micrograph shows, size of the obtained CuO NPs was in 10–60 nm size range.

The EDX study of the chemical composition of CuO NPs confirms that Cu was observed at an atomic percentage of 73.8%, the other elements carbon, oxygen, and sodium were detected in the EDX spectra with an atomic percentage of 14.4, 9.7, and 2.1%, respectively. The results confirm that the NPs are formed with Cu and O. The presence of carbon and oxygen in the NPs originated from the bioactive chemical constituents present in the aqueous extract of *S. maritima*. The atomic % of copper was found to be high. Oxygen composition was low than the carbon and less composition of sodium was detected in the EDX spectra. The detection of sodium in the EDX spectra may be origin from the plant which is grown in the mangrove soils and salt is deposited in the plant tissues. Polydispersity index (PDI) is the parameter used to indicate the uniformity and the homogeneity of the NPs and PDI of less than 0.2 is preferred for monodispersity. In the present study, the PDI of the NPs synthesized using *S. maritima* aqueous plant extract was found to be 1.593 which corroborated the monodispersity of the NPs. In the XRD spectra, no peaks corresponding to other phases detected confirm that CuO NPs were pure and other phases were involved in the structure. The Debye-Scherrer equation [33] was utilized for evaluation of crystallite size of the synthesized CuO NPs and the average size obtained was 34 nm which is in good correlation with the results observed in particle size distribution.

The extract and the synthesized CuO NPs show antibacterial activity against the bacterial strains studied. Aqueous plant extract at 1 μg/mL concentration shows no zone of inhibition for *E. coli* and *P. aeruginosa* whereas in same concentration prominent zones were observed for CuO NPs. The zone of inhibition in all the studied concentration was found to be more for synthesized CuO NPs than water extract and no zone of inhibition observed for control.

In photocatalytic degradation of methylene blue, it was observed that more and more dye molecules were adsorbed on the surface of the photocatalyst, when initial concentration of the dye was increased. Because many active sites were occupied by the dye molecules, the adsorption of O_2_ and OH^−^ on the photocatalyst was decreased, which leads to reduced generation of radicals. Furthermore, the photons were blocked before reaching the photocatalyst surface; hence, the adsorption of photons was decreased by the photocatalyst. Accordingly, the removal rate reduced at high initial dye concentrations. The photocatalytic degradation of methylene blue results confirms that % degradation with time was found to be more efficient in the present study and are in correlation with the finding reported [34, 35].

The NPs synthesized using chemical synthesis have limitations such as stability in hostile environment, lack of understanding in fundamental mechanism and modeling factors, bioaccumulation/toxicity features, expansive analysis requirements, need for skilled operators, problem in devices assembling and structures, and recycle/reuse/regeneration. But the present study is the green synthesis approach and NPs produced through regulation, control, clean up, and remediation process will directly help uplift their environmental friendliness.

## Conclusions

The study highlighted the utilization of *S. maritima* (L.) Dumort in the domain of nanotechnology and synthesized CuO NPs. The FT-IR spectra of CuO NPs shows the presence of bioactive functional groups that are present in the plant extracts are the responsible for the bio-capping of the CuO NPs. The synthesized CuO NPs were characterized and confirms that the particle size of around 34 nm having circular in shape with rough surface morphology. The synthesized CuO NPs were found to be potent against growth of common microbial pathogens and are also having DPPH radical inhibition antioxidant activity. The synthesized CuO NPs exploited as photocatalyst exhibited excellent degradation efficiency methylene blue dye. The % degradation of dye was higher than the reported methods and was confirmed to be advantaged than the reported. Thus, the presented method is quick, convenient, environment-friendly, nontoxic, and free from organic solvents, surfactants, and specialized instruments.

## Data Availability

Not applicable.

## Abbreviations

CuO NPs: Copper oxide nanoparticles
CuO: Copper oxide
NPs: Nanoparticles
UV-Vis: UV-visible spectrophotometry
XRD: X-ray diffraction
FT-IR: Fourier transform infrared spectroscopy
SEM: Scanning electron microscopy
